# Phenylethyl isothiocyanate mitigates drug-induced liver injury in mice by inhibiting hepatocyte pyroptosis through the NLRP3-caspase-1-GSDMD pathway

**DOI:** 10.3389/fphar.2025.1539934

**Published:** 2025-03-25

**Authors:** Ning An, Xiaoru Wang, Jia Qin, Meng Cheng, Mei Bai, Jingcai Cheng, Qiang Xu, Xuefeng Wu

**Affiliations:** ^1^ State Key Laboratory of Pharmaceutical Biotechnology, Nanjing Drum Tower Hospital, School of Life Sciences, Nanjing University, Nanjing, China; ^2^ Drug R&D Institute, JC (Wuxi) Company, Inc., Wuxi, China

**Keywords:** liver injury, triptolide, phenylethyl isothiocyanate, pyroptosis, GSDMD

## Abstract

**Introduction:** Drug-induced liver injury (DILI) represents a distinct form of hepatic damage resulting from exposure to pharmacological agents. The pathological mechanisms underlying DILI are multifaceted and remain incompletely elucidated. However, emerging evidence suggests that cell pyroptosis, a form of programmed cell death associated with inflammation, may serve as a common mechanistic pathway in DILI pathogenesis.

**Methods:** To investigate the role of pyroptosis in DILI, we established a murine model of DILI using triptolide and evaluated the therapeutic potential of phenethyl isothiocyanate (PEITC), a naturally occurring compound, in mitigating liver injury through the modulation of hepatocyte pyroptosis. Mice were administered PEITC at doses ranging from 5 to 20 mg/kg. Cytokine expression was measured using quantitative polymerase chain reaction or biochemical indicator analyzer. Cell signalings were assayed by western blot and immunohistochemistry. The AML12 hepacytes were cultured to investigate the *in vitro* effects.

**Results:** PEITC treatment markedly attenuated hepatic tissue damage, restored normal liver architecture, and significantly reduced serum levels of transaminases (AST and ALT), while normalizing hepatic metabolic function. These protective effects were mechanistically linked to the suppression of hepatocyte pyroptosis, as PEITC effectively reversed the upregulation of the NLRP3 inflammasome, Caspase-1 cleavage, and Gasdermin D (GSDMD) in triptolide-exposed livers. *In vitro* studies using cultured hepatocytes further demonstrated that PEITC inhibited the expression and activation of NLRP3, Caspase-1, GSDMD, and other key proteins involved in the pyroptosis pathway. Ultrastructural analysis via electron microscopy corroborated these findings, revealing that PEITC prevented pyroptosis-induced membrane pore formation in hepatocytes.

**Conclusions:** PEITC exerts hepatoprotective effects against DILI by targeting the pyroptosis pathway, thereby highlighting its potential as a novel therapeutic strategy for liver injuries. Our results further implicate cell pyroptosis as a novel target for the attenuation of DILI.

## Introduction

The liver serves as the primary organ for drug metabolism and is consequently subject to the highest levels of toxicity from various medications, whether derived from the original compounds or their metabolites. The metabolism of numerous drugs *in vivo*, particularly those that require prolonged administration, has been associated with a range of adverse effects, most notably liver damage. While certain drug-related liver injuries are benign and may resolve upon discontinuation of the medication, an increase in drug-induced liver injury (DILI) can lead to chronic liver conditions or, in severe cases, acute liver failure (Zhang et al., 2023; You et al., 2018).

DILI can arise from a wide array of medications, including prescription drugs, over-the-counter medications, herbal remedies, and, occasionally, recreational substances. Generally, DILI is classified into two categories: intrinsic and idiosyncratic types (Huang et al., 2019). Intrinsic toxicity can be reliably replicated in animal studies and is characterized by a dose-dependent relationship, even at sublethal concentrations. Furthermore, environmental factors and genetic predispositions may influence the toxicity associated with intrinsic hepatotoxicants (Cai et al., 2021).

Triptolide is a bioactive compound derived from the traditional Chinese medicinal herb known as *Tripterygium wilfordii*. This compound shows a range of distinct pharmacological effects, which make it especially significant in both research and therapeutic applications. However, it is important to look out that triptolide is also associated with considerable adverse effects, particularly concerning liver toxicity. Research has shown that triptolide can induce a form of programmed cell death called PANoptosis in macrophages *in vitro*. Furthermore, triptolide has exhibited both nephrotoxic and hepatotoxic effects that correlate with its ability to induce PANoptosis (Zhang et al., 2023). It disrupts mitochondrial function, reducing adenosine triphosphate (ATP) production and causing mitochondrial membrane depolarization. This dysfunction leads to energy depletion in liver cells, contributing to hepatocyte death through apoptosis or necrosis (You et al., 2018). Triptolide also causes the body to consume large amounts of xanthine and uric acid, causing the generation of various reactive oxygen species (ROS) due to oxidative stress, stimulates the signaling pathway of toll-like receptors and enhances the phosphorylation of the downstream protein nuclear factor κ-light-chain-enhancer of activated B cells (NF-κB), which induces inflammation and eventually results in cellular dysfunction and apoptosis (Huang et al., 2019; Cai et al., 2021). Recent studies suggest that pyroptosis, a form of inflammatory cell death, plays a crucial role in triptolide-induced liver injury. Triptolide may activate inflammasomes like nucleotide-binding oligomerization domain-like receptor protein 3 (NLRP3), leading to Caspase-1 activation, Gasdermin D (GSDMD) cleavage, and subsequent pyroptotic liver cell death. This process is connected to the release of inflammatory cytokines, further exacerbating liver inflammation and injury (Cai et al., 2021). Pyroptosis also occurs in thioacetamide- and acetaminophen-induced liver damage accompanied by intense oxidative stress and inflammation (Chen et al., 2016). Therefore, triptolide may be a good compound to induce a DILI model, particularly in experimental settings where researchers aim to study mechanisms of liver damage, toxicity, and inflammation. Investigating the pyroptosis process induced by triptolide may advance treatment strategies for a range of drug-induced liver injuries.

Phenethyl isothiocyanate (PEITC) is one of the major bioactive compounds present in cruciferous vegetables (such as watercress, broccoli, and sprout kale). Owing to the presence of electron-withdrawing ITC groups (N=C=S), PEITC is highly reactive with cysteine, leading to multiple intracellular targets. PEITC influences crucial cellular signaling routes associated with the advancement of cancer, notably the NF-κB, protein kinase B (Akt), and mitogen-activated protein kinase (MAPK) pathways, which play a role in activating B cells (Wang et al., 2011; Cavell et al., 2012). It can alter the cytoskeletal structure and epigenome of bacterial cells, and induce the unfolded protein response (UPR) and autophagy (Dayalan Naidu et al., 2018). Additionally, it has been demonstrated that PEITC activates the cytoprotective pathway facilitated by the transcription factor Nrf2 (nuclear factor erythroid 2-related factor 2) and the heat shock response (HSR) controlled by heat shock factor 1 (HSF1), while also inhibiting phase 1 enzymes when present in elevated concentrations. It is also a potential natural nutraceutical with bioactive structure that promote gastrointestinal health (Gupta et al., 2014). Our earlier research has demonstrated that PEITC protects the liver (Wang et al., 2022). Thus, we examined alterations of protein expression, formation of pores in the cell membrane and process of pyroptosis in livers from mice treated with triptolide. We further tested whether and how PEITC could alleviate the drug-induced injury both *in vivo* and *in vitro*.

## Materials and methods

### Chemicals and reagents

Phenethyl isothiocyanate (PEITC) was obtained from JC (Wuxi) Company, Inc. (Wuxi, China), while triptolide was provided by Acmec Biochemical Technology (Shanghai, China). Alexa Fluor 488-conjugated anti-rabbit IgG and Dulbecco’s Modified Eagle’s Medium/Nutrient Mixture F-12 (DMEM/F12) were sourced from Thermo Fisher Scientific (Waltham, MA, United States). The ChamQ Universal SYBR qPCR Master Mix and HiScript III RT SuperMix for quantitative polymerase chain reaction (qPCR) were supplied by Vazyme, a reputable biotechnology company located in Nanjing, China. Additional reagents for cell lysis, essential for techniques such as Western blotting and immunoprecipitation, were procured from Beyotime Biotechnology (Shanghai, China). Insulin-transferrin-selenium (ITS) media supplements were provided by R&D Systems (Minneapolis, MN, United States). Antibodies targeting α-tubulin were sourced from Abmart (Shanghai, China), while the antibody for Nod-like receptor family pyrin domain containing 3 (NLRP3) was obtained from Cell Signaling Technology (Danvers, MA, United States). Anti-caspase-1 and anti-IL-1β antibodies were acquired from Santa Cruz Biotechnology (Santa Cruz, CA, United States), and the anti-GSDMD antibody was procured from Abcam (Cambridge, United Kingdom). Bovine Serum Albumin (BSA) was were procured from Sangon Biotech (Shanghai, China).

### Animal experiments

ICR mice (6–8 weeks old) were purchased from SPF (Beijing) Biotechnology Company, Inc. and housed in an SPF-grade animal facility maintained on a 12:12 h light/dark cycle, with ambient temperatures ranging from 22°C to 25°C. They had unrestricted access to food and sterile water. Based on references (Wang et al., 2023; Yang et al., 2012) and preliminary experimental results, a model of drug-induced liver injury was established through the oral administration of Triptolide at a dosage of 0.75 mg/kg once daily for two consecutive days. PEITC was carefully diluted in olive oil to the desired concentration for oral administration. Twenty-five male mice were randomly assigned to five groups: control, Triptolide treatment, Triptolide + 5 mg/kg PEITC, Triptolide + 10 mg/kg PEITC, and Triptolide + 20 mg/kg PEITC. Female mice were grouped identically to the males. Mice in each group received varying concentrations of PEITC 3 days prior to the administration of triptolide, while those in the normal control or Triptolide-treated group also received equivalent volume of olive oil (10 mL/kg) at the same time. The final Triptolide dose was given 24 h prior to serum collection, and thereafter, the mice were euthanized. The livers were harvested, weighed, and photographed for documentation. A portion of the liver right lobe tissue was fixed in paraformaldehyde, embedded in paraffin, sectioned, and the remaining tissue was stored at −80°C (Wang et al., 2022).

### Hematoxylin-eosin (HE) staining and histological activity index (HAI)

Following fixation of liver tissue with paraformaldehyde, the samples were processed by Wuhan Servicebio Co., Ltd. (Wuhan, China) for paraffin embedding and sectioning, followed by HE staining. The HAI scoring system was utilized to evaluate hepatic inflammation and necrosis in HE-stained sections. HAI, a cumulative score of liver inflammation reflecting lobular, periportal, portal inflammation, and fusion necrosis, ranges from 0 to 18, with scores of 7 or higher indicating significant necroinflammation.

### Analysis of serum biochemical indicators

Hitachi 7600-020 automatic biochemistry analyzer was employed to measure the serum concentrations of aspartate aminotransferase (AST), alanine aminotransferase (ALT), γ-glutamyl transpeptidase (GGT), lactate dehydrogenase (LDH), triglycerides (TG), total cholesterol (CH), direct bilirubin (DBIL), and albumin (ALB).

### Real-time PCR

RNA was extracted from liver tissues using TRIzol reagent, following the manufacturer’s protocol. The extracted RNA was reverse-transcribed into cDNA according to the same TRIzol protocol. The amplification conditions were as follows: an initial step at 95°C for 2 min (1 cycle), followed by 45 cycles of denaturation at 95°C for 10 sec, annealing at 60°C for 30 sec, and extension at 95°C for 10 sec. The primers used in this study were shown in Supplementary Table.

### Western blot

Immunoblotting was performed essentially as described previously (Wang et al., 2023). Briefly total protein extraction from mouse liver tissue was conducted using RIPA buffer supplemented with the protease inhibitor 1% phenylmethylsulfonyl fluoride (PMSF). Liver tissues were lysed in this buffer, and after 30 min of incubation on ice, the supernatant containing the extracted proteins was collected via centrifugation at 12,000 × g for 15 min. Protein concentrations were determined using the BCA protein assay kit, with bovine serum albumin (BSA) as the standard. The total protein samples were resolved using 10% Tris-glycine SDS-PAGE and subsequently transferred to a polyvinylidene fluoride (PVDF) membrane. The primary antibody was diluted to a working liquid with 3% BSA (NLRP3, 1:1,000; Caspase-1, 1:1,000; GSDMD, 1:1,000; IL-1β, 1:1000; Tubulin, 1:4,000). Various antibodies were employed for incubation at 4°C for 12 h, based on the molecular weight of the target proteins.

### Immunohistochemistry

Immunofluorescence analysis was performed as previously described (Wang et al., 2023). Liver specimens underwent dewaxing with xylene, followed by dehydration through a gradient ethanol series to facilitate antigen retrieval in a water bath at 95°C for 15 min using citrate buffer (pH 6.0). Sections were treated with 0.3% H2O2 for 7 min to eliminate endogenous peroxidase activity and then incubated with 2% BSA for 30 min to block non-specific binding sites. The slices were incubated overnight at 4°C with primary antibodies (NLRP3, 1:200; Caspase-1, 1:100; GSDMD, 1:200; IL-1β, 1:200). After washing with phosphate-buffered saline (PBST), sections were stained according to the instructions provided with the anti-mouse/rabbit universal immunohistochemical detection kit, followed by counterstaining with hematoxylin.

### Cell culture

AML12 cells were obtained from the National Collection of Authenticated Cell Cultures (Shanghai, China). These cells were cultured in DMEM/F12 medium supplemented with 10% fetal bovine serum (FBS) and an ITS solution at a final concentration of 1x, along with 40 ng/mL of dexamethasone. A penicillin-streptomycin solution at 1x concentration was also added to minimize bacterial contamination. The cells were maintained in an incubator with a controlled environment of 5% (v/v) carbon dioxide and 37°C. To assess the effects of PEITC on the drug-induced liver injury model, AML12 cells were treated with PEITC (0.1, 0.3, 1, or 3 µM) for 1 h and subsequently with 40 nM Triptolide for 24 h. The cells were then collected for further experiments.

### Immunofluorescence assay

AML12 cells underwent a series of preparatory steps, including fixation, permeabilization, and blocking, to facilitate effective antibody binding. Following these preparations, the cells were incubated overnight at 4°C with anti-GSDMD antibodies diluted with 3% BSA [1:100]. After extensive washing with phosphate-buffered saline (PBS) to remove unbound antibodies, the cells were treated with Alexa Fluor 488-conjugated anti-rabbit IgG for visualization. The nuclei were stained using DAPI, a fluorescent dye that binds to DNA, providing clear contrast for examining cellular structures under microscopy.

### Electron microscopy

Cell samples were fixed using standard protocols for electron microscopy (Servicebio, Wuhan, China) and subsequently processed for scanning electron microscopy at Wuhan Servicebio Co., Ltd. (Wuhan, China).

### Statistical analysis

Results are expressed as mean ± SEM, with each experiment performed in triplicate. Statistical analyses were conducted using GraphPad Prism 8 software (San Diego, CA, United States). Data were analyzed using Student’s *t*-test or one-way analysis of variance (ANOVA), followed by Bonferroni *post hoc* correction. A *P*-value of <0.05 was considered statistically significant.

## Results

### PEITC ameliorated acute drug-induced liver injury in mice caused by triptolide

**FIGURE 1:**
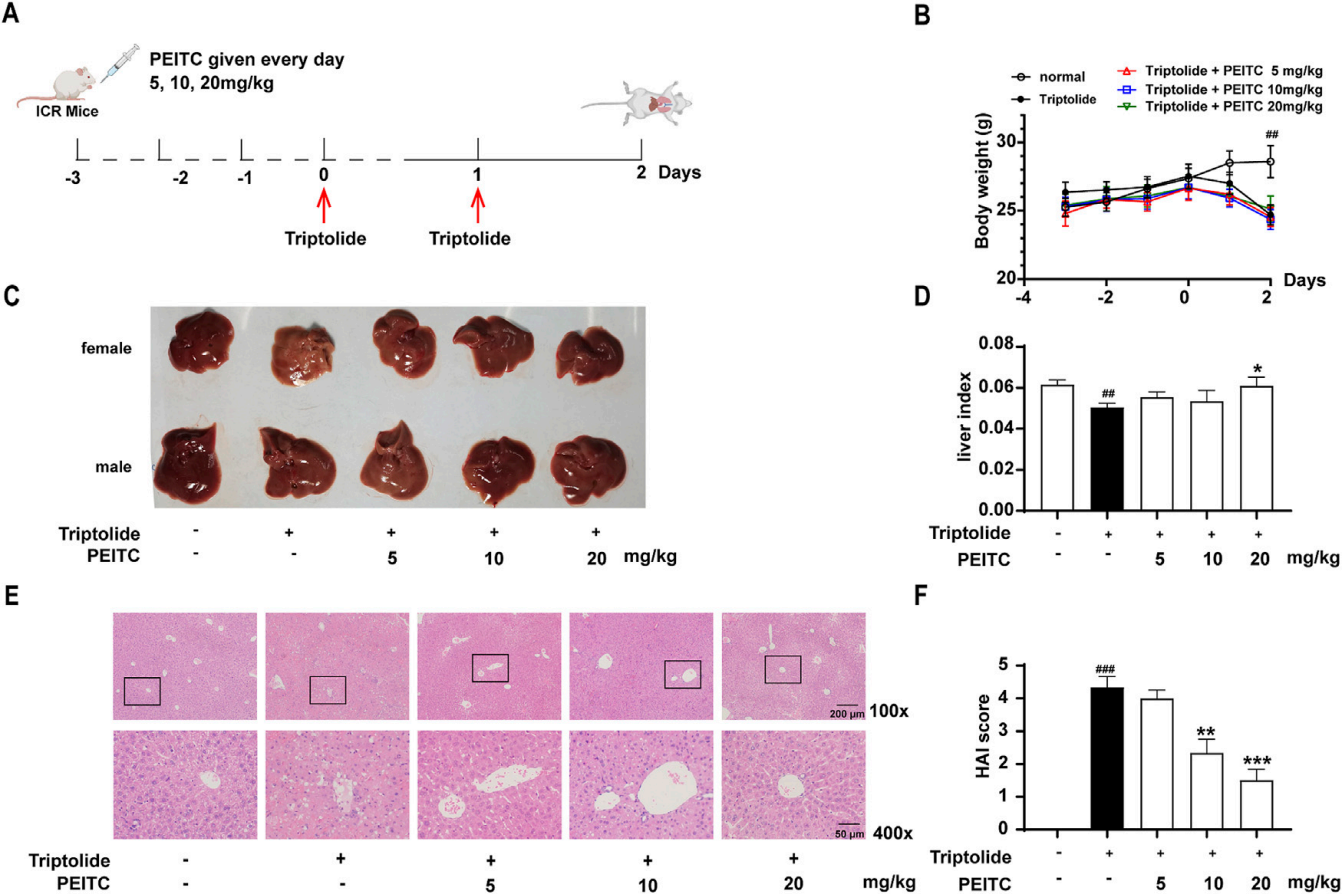
PEITC ameliorated acute drug-induced liver injury in mice caused by triptolide. **(A)** Schematic of the triptolide-induced acute drug-induced liver injury model. **(B)** Body weight changes over time. **(C)** The lesions on the surface of the liver tissues were photographed and recorded. **(D)** Liver indices were measured. **(E)** Representative images of hepatic histology. All samples examined are from the right lobe of the liver tissue. Scale bar, 100 μm. **(F)** HAI scores of the liver histopathology. The data are presented as the means ± SEMs (*n* = 10 per group). ^##^ *P* < 0.01 vs. the normal group.

Based on the experimental schematic presented in Figure 1A, a drug-induced liver injury model was established by administering triptolide to mice via intragastric gavage at a dosage of 0.75 mg/kg once daily for two consecutive days. In each experimental group, varying concentrations of phenethyl isothiocyanate (PEITC) were administered orally to the PEITC-treated mice, while the triptolide-treated mice received a specified volume of solvent (olive oil), beginning 3 days prior to triptolide administration and continuing daily until the end of the study. The mice were euthanized 24 h after the final triptolide administration, and their livers were excised and photographed.

During the initial 3 days of oral PEITC administration, no significant differences in body weight were noted among the various groups of mice (Figure 1B). However, following triptolide treatment, a marked reduction in body weight was observed. The livers of the triptolide-treated mice appeared smaller and exhibited a whitish surface compared to those in the control group. In contrast, PEITC treatment resulted in improved liver morphology across all experimental groups (Figures 1C,D). Histological examination using H&E staining revealed distinct hepatic cords radiating from the central vein in the livers of the normal control group, where hepatocytes were closely packed, uniform in size, and retained normal structural characteristics. Conversely, triptolide-treated mice displayed pronounced pathological changes, including cell necrosis, structural distortions, disorganized hepatic cords, indistinct boundaries of the central portal vein, and inflammatory cell infiltration. However, the liver morphology of mice treated with PEITC showed dose-dependent restoration, with notable improvements toward normalcy (Figure 1E). Additionally, histopathological analysis scores of H&E-stained liver tissue sections indicated that PEITC treatment significantly reduced liver pathology scores compared to the triptolide group (Figure 1F). These findings suggest that PEITC markedly ameliorates liver tissue pathology in mice experiencing acute drug-induced liver injury induced by triptolide.

### PEITC reduced hepatic biochemical indices in mice with triptolide-induced liver injury

**FIGURE 2:**
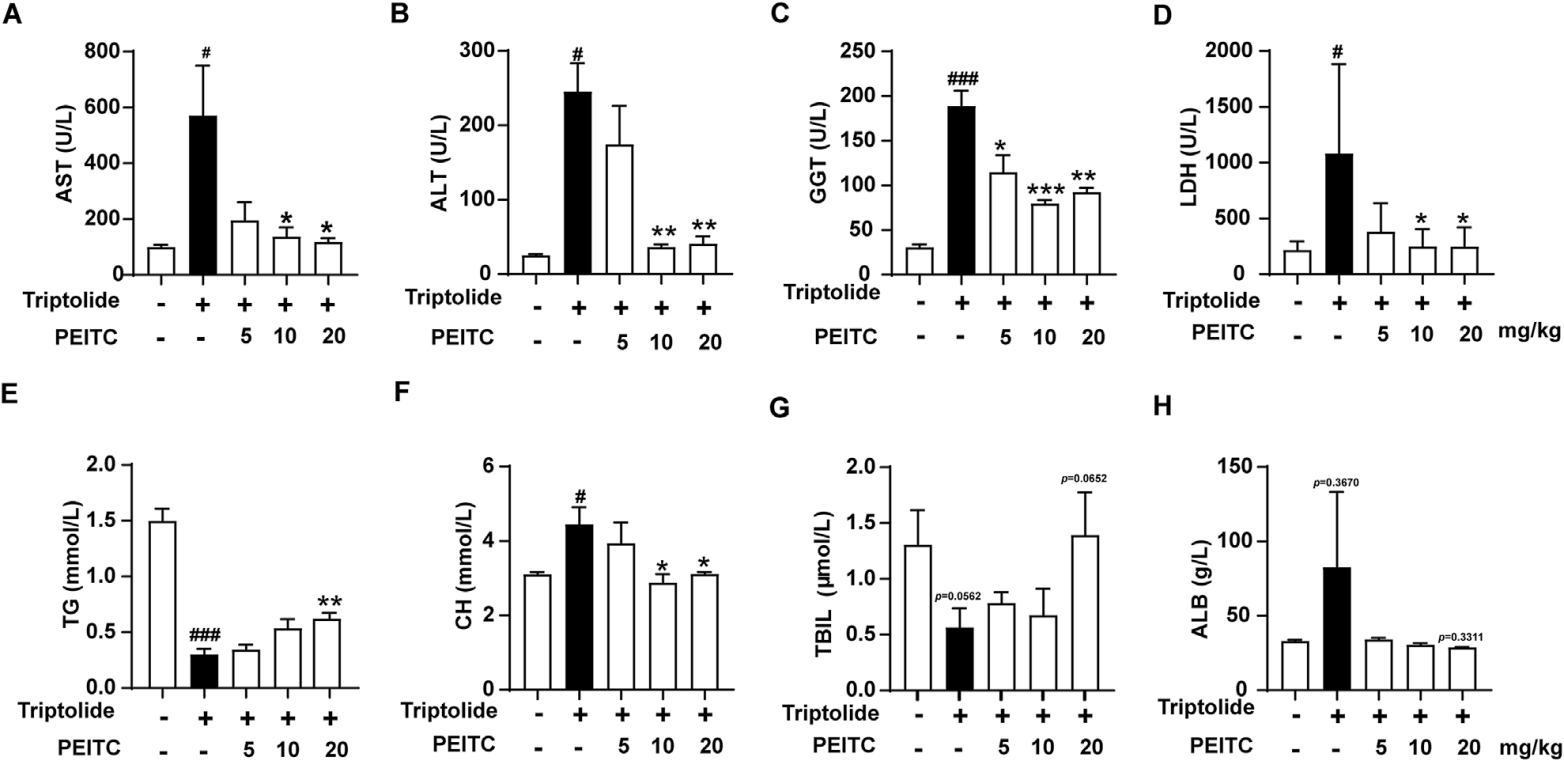
PEITC improved hepatic biochemical indices. **(A)** Serum AST levels. **(B)** Serum ALT levels. **(C)** Serum GGT levels. **(D)** Serum LDH levels. **(E)** Serum TG levels. **(F)** Serum CH levels. **(G)** Serum TBIL levels. **(H)** Serum ALB levels. The data are presented as the means ± SEMs (n = 6 per group). #*P* < 0.05, ###*P* < 0.001 vs. the normal group; **P < 0.05, **P < 0.01, *P* < 0.001 vs. the triptolide control.

Transaminase activity levels in serum are widely recognized as indicators of liver cell damage and are considered highly sensitive and specific biomarkers for hepatotoxicity. Our study focused on measuring the levels of key liver biochemical markers-AST, ALT, GGT, and LDH-in the various treatment groups of mice. We observed that PEITC effectively inhibited the increase in serum levels of AST, ALT, GGT, and LDH in mice treated with triptolide (Figures 2A–D).

Additionally, we analyzed the serum levels of triglycerides (TG), cholesterol (CH), total bilirubin (TBIL), and albumin (ALB) across the different treatment groups. Our findings revealed that in the triptolide-treated group, cholesterol levels increased, while total bilirubin and triglycerides decreased (Figures 2E–G). These results suggest that cholesterol metabolism in the liver is impaired, resulting in reduced bile synthesis and compromised triglyceride digestion and absorption. In contrast, treatment with PEITC led to varying degrees of improvement in these metabolic indicators. Albumin (ALB), a critical protein in the blood, typically decreases in the context of liver dysfunction and malnutrition. However, in the cases of triptolide-induced acute liver injury observed in our study, serum albumin levels exhibit an increase (Figure 2H). This phenomenon may be related to compensatory synthesis. In the early stages of acute injury, liver damage may not be severe in some animals, allowing the liver to compensate by increasing the synthesis of albumin to counteract losses due to other factors, such as the need for enhanced transport functions or inflammatory reaction. A comparison of serum ALB levels among the different groups demonstrated that PEITC mitigated the change in serum ALB levels caused by triptolide treatment.

Overall, these findings demonstrate that PEITC offers protective effects against acute drug-induced liver injury and contributes to the restoration of normal liver metabolic function.

### PEITC prevented hepatocyte pyroptosis in mice with triptolide-induced liver injury

Hepatocyte death is a significant mechanism underlying liver injury. The objective of this study was to determine whether PEITC can directly prevent hepatocyte death. Given PEITC’s well-documented anti-inflammatory properties and the role of pyroptosis as a form of cell death closely associated with inflammatory responses, particularly in the context of acute drug-induced liver injury, we investigated the significance of pyroptosis in liver damage.

**FIGURE 3:**
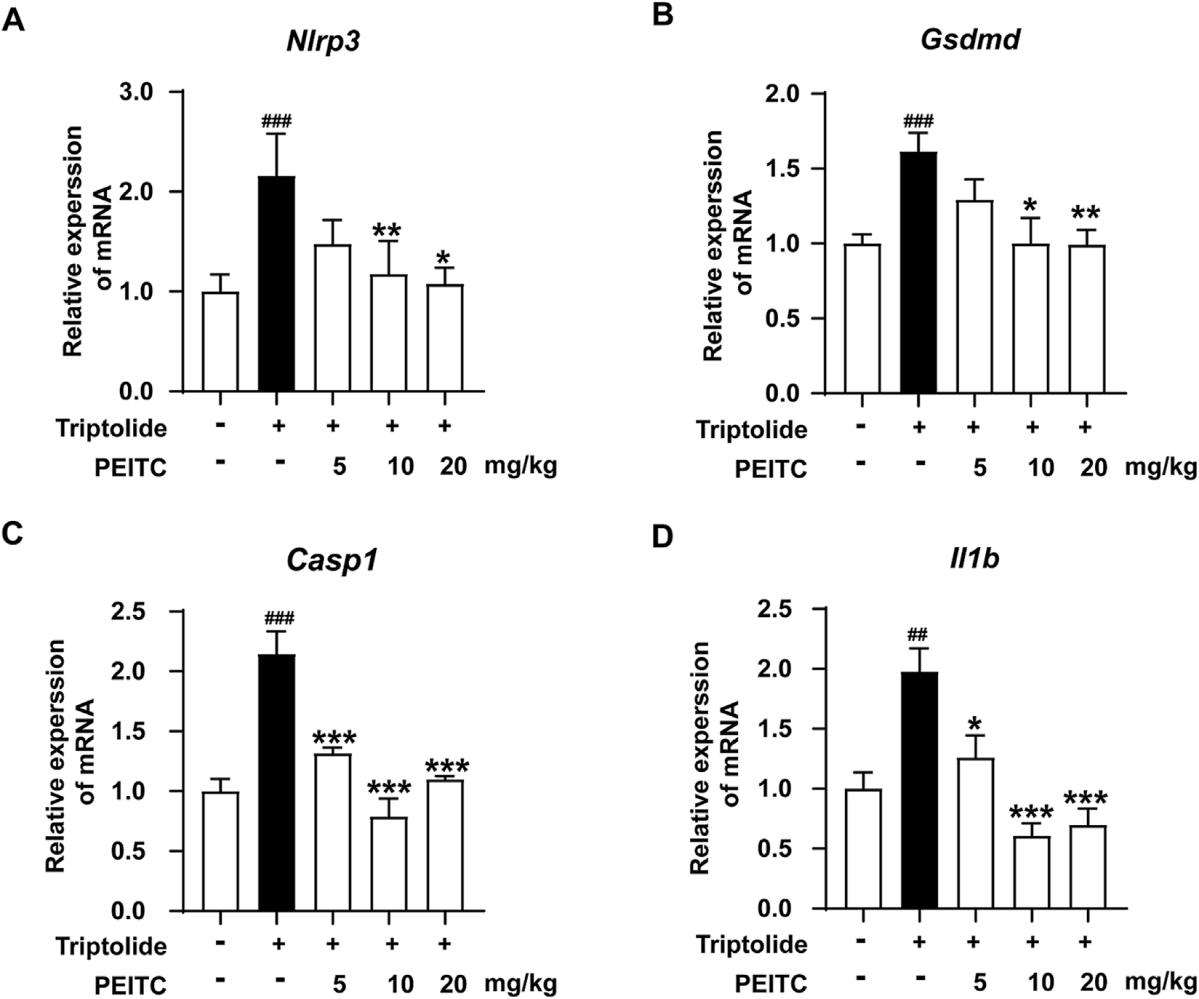
PEITC reduced the mRNA levels of pyroptosis-related proteins in mice with liver injury induced by triptolide. **(A)** Relative expression of *Nlrp3* mRNA in liver tissues. **(B)** Relative expression of *Casp1* mRNA in liver tissues. **(C)** Relative expression of *Gsdmd* mRNA in liver tissues. **(D)** Relative expression of *Il1b* mRNA in liver tissues. The data are presented as the means ± SEMs (n = 6 per group). ^##^ *P* < 0.01, ^###^ *P* < 0.001 vs. the normal group; ^**^ *P* < 0.05, ^**^ *P* < 0.01, ^**^ *P* < 0.001 vs. the triptolide control.

Analysis using quantitative PCR (qPCR) revealed that the mRNA expression levels of pyroptosis-related proteins *Nlrp3, casp1, Gsdmd,* and *Il-b* were significantly elevated in the liver tissue of mice treated with triptolide. In contrast, PEITC treatment resulted in reduced mRNA expression levels of these pyroptosis-related proteins in mice (Figure 3).

**FIGURE 4:**
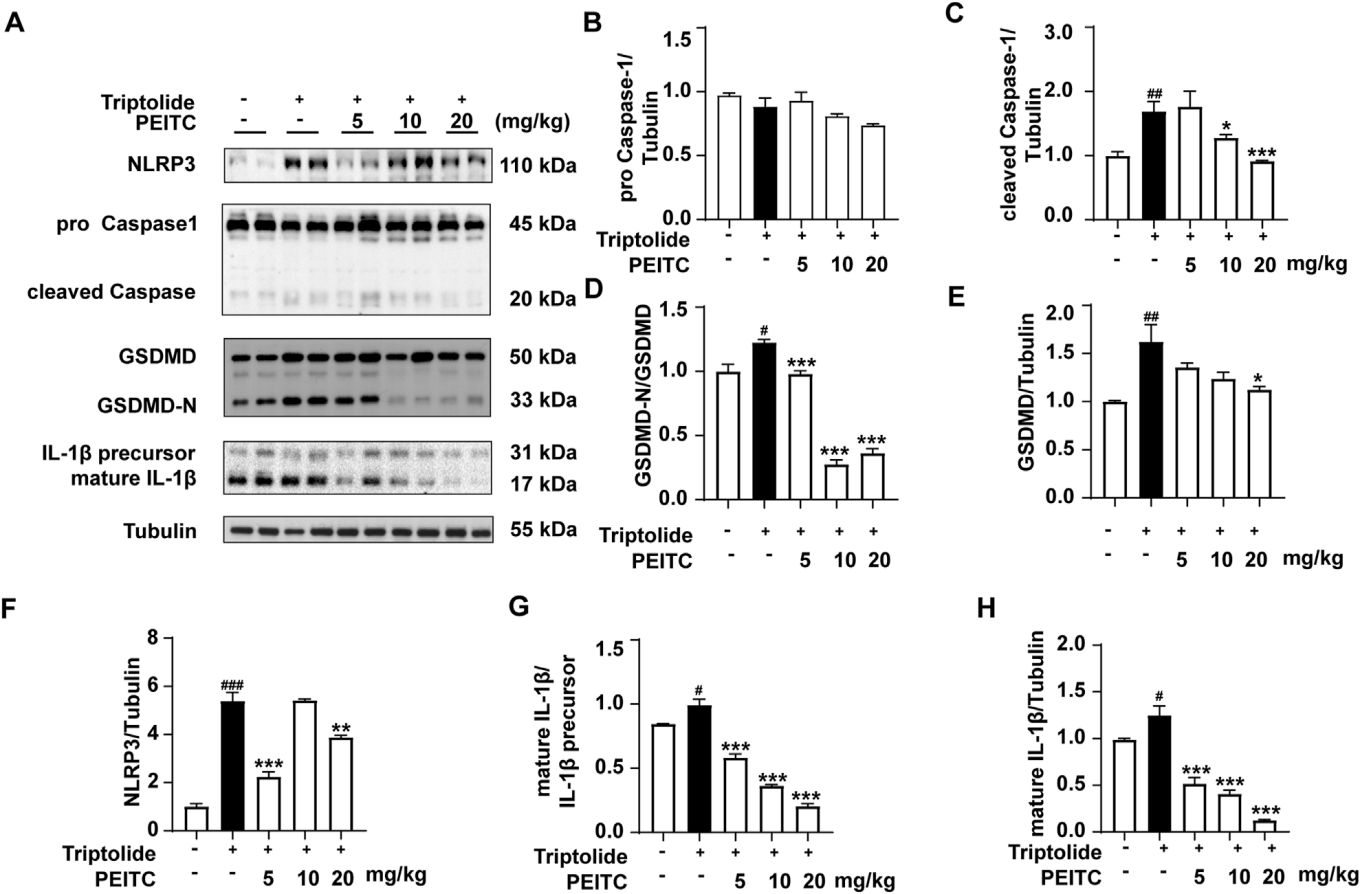
PEITC prevented hepatocyte pyroptosis in mice with liver injury induced by triptolide. **(A)** Immunoblot analysis of NLRP3, pro-Caspase-1, cleaved Caspase-1, GSDMD, GSDMD-N, the IL-1β precursor, mature IL-1β and Tubulin. **(B–H)** The relative protein levels of pro-Caspase-1/Tubulin, cleaved Caspase-1/Tubulin, GSDMD-N/GSDMD, GSDMD/Tubulin, NLRP3/Tubulin, the IL-1β/IL-1β precursor, and mature IL-1β/Tubulin. The data are presented as the means ± SEMs (*n* = 6 per group). ^##^ *P* < 0.01, ^###^ *P* < 0.001 vs. the normal group; ^**^ *P* < 0.05, ^**^ *P* < 0.01, ^**^ *P* < 0.001 vs. the triptolide control.

In addition, Western blotting was conducted to confirm the expression levels of the pyroptosis-related proteins NLRP3, pro-Caspase-1, cleaved Caspase-1, GSDMD, GSDMD-N, and IL-1β in the livers of the mice treated with triptolide. The results revealed an increase in the expression of NLRP3, as well as increased cleavage and activation of the pyroptosis-related proteins caspase-1, GSDMD, and il-1β, comparable to those in the normal group. However, after PEITC treatment, there was a significant reduction in the expression of NLRP3 and the levels of cleaved Caspase-1, GSDMD-N and mature IL-1β (Figure 4).

**FIGURE 5:**
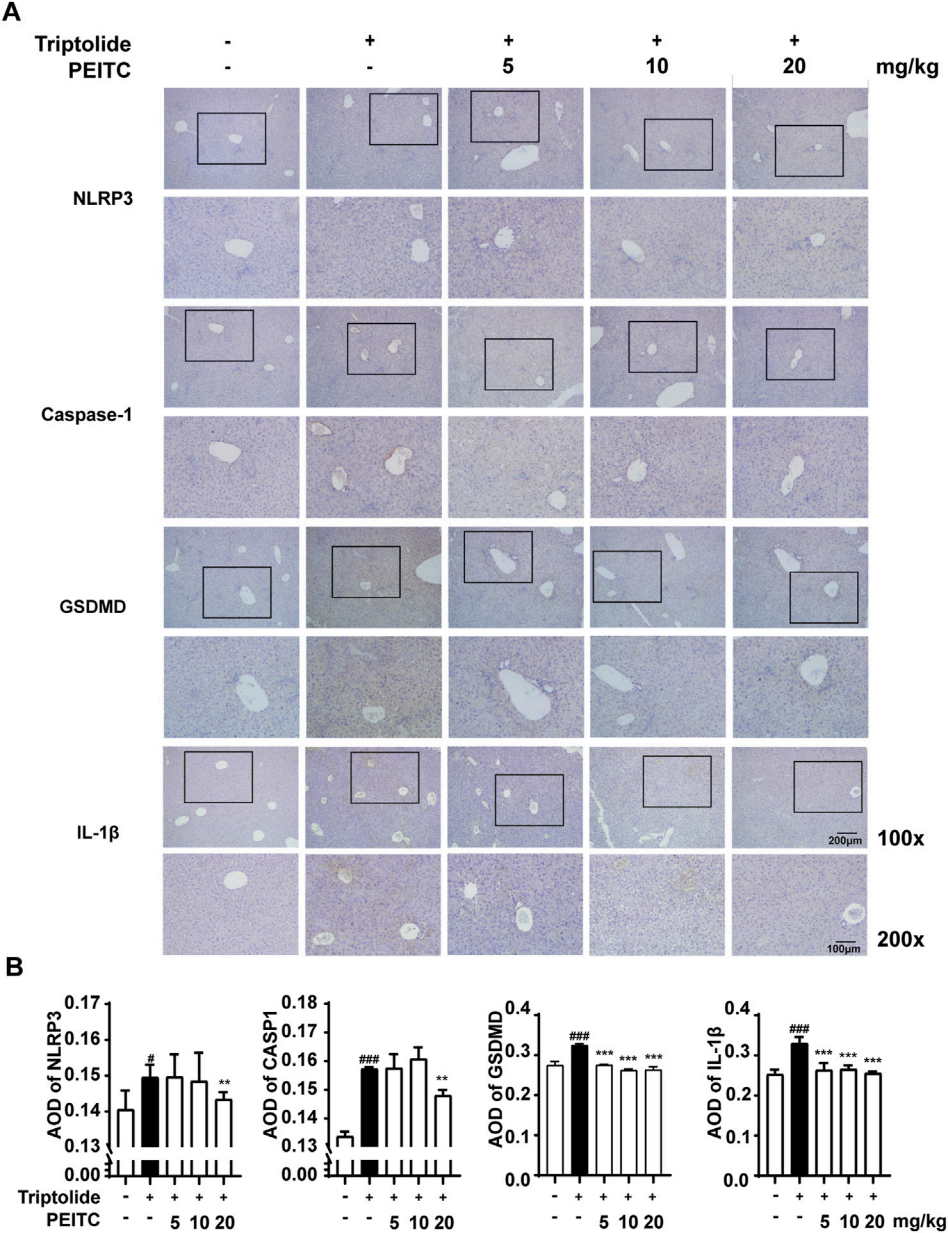
PEITC reduced the expression levels of pyroptosis-related proteins. **(A)** Liver tissue sections from each group were stained for NLRP3, Caspase-1, GSDMD, and IL-1β. Scale bar, 100 μm. **(B)** The average optical densities (AODs) of NLRP3, Caspase-1, GSDMD, and IL-1β are displayed as histograms. The data are presented as the means ± SEMs (n = 10 per group). ##*P* < 0.01, ###*P* < 0.001 vs. the normal group; **P < 0.05, **P < 0.01, *P* < 0.001 vs. the triptolide control.

Next, we performed immunohistochemical analysis on liver sections from the mice in each group. The immunohistochemistry results revealed that the expression levels of NLRP3, cleaved caspase-1, GSDMD (GSDMD and GSDMD-N), and IL-1β were greater in the triptolide-treated group than in the normal group (Figures 5A–E). PEITC inhibited the expression of NLRP3, cleaved caspase-1, GSDMD (GSDMD and GSDMD-N) and mature IL-1β in a dose-dependent manner (Figure 5). These findings indicate that the improvement of DILI by PEITC is indeed accompanied by significant inhibition of cell pyroptosis process. However, whether this effect directly affects hepatocytes will be demonstrated later through *in vitro* experiments.

### PEITC effectively inhibited pyroptosis in AML12 cells treated with triptolide *in vitro*

**FIGURE 6:**
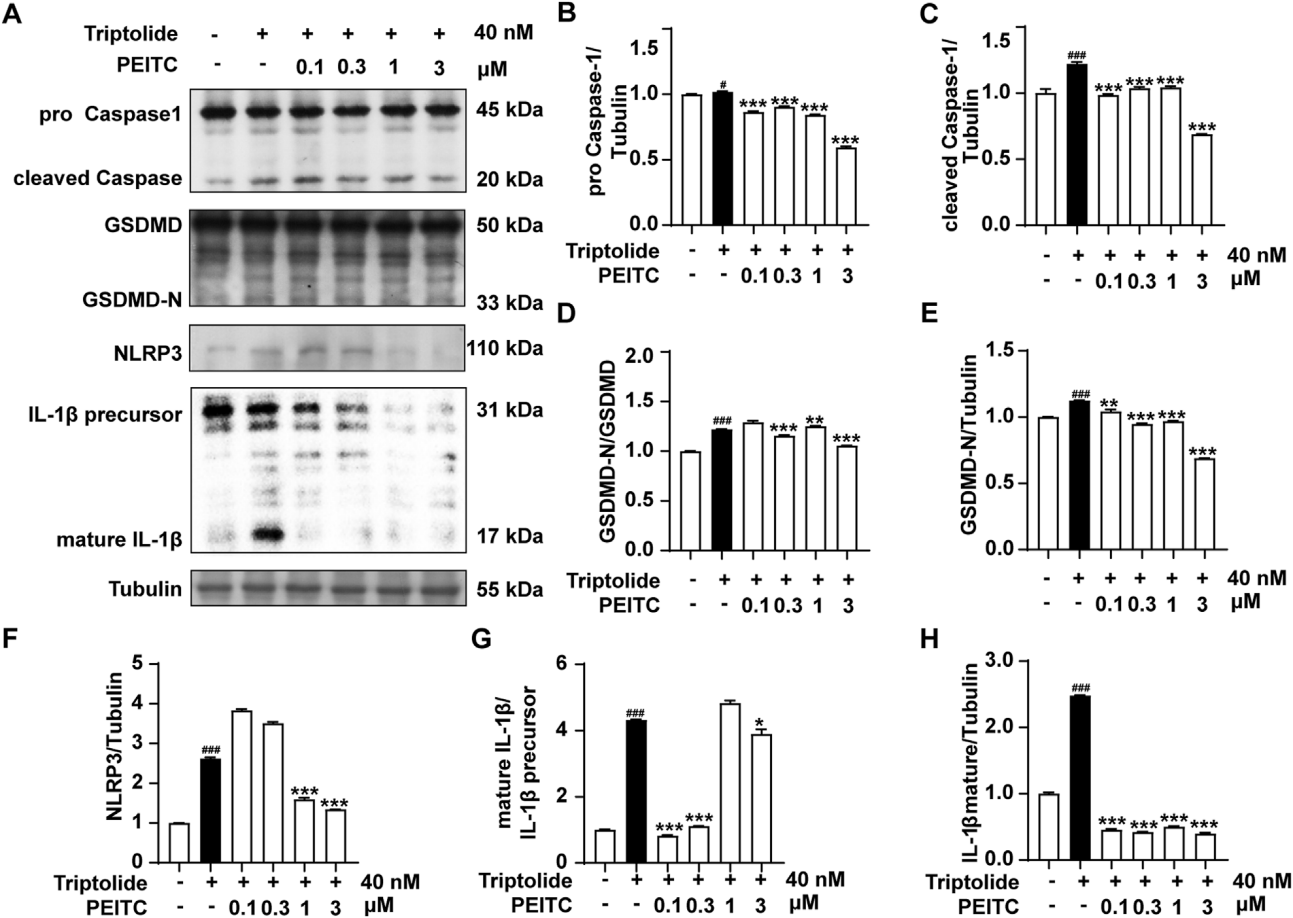
PEITC prevented AML12 cell pyroptosis induced by triptolide *in vitro*. **(A)** Immunoblot analysis of NLRP3, pro-Caspase-1, cleaved Caspase-1, GSDMD, GSDMD-N, the IL-1β precursor, mature IL-1β and Tubulin. **(B–H)** The relative protein levels of pro-Caspase-1/Tubulin, cleaved Caspase-1/Tubulin, GSDMD-N/GSDMD, GSDMD/Tubulin, NLRP3/Tubulin, the IL-1β/IL-1β precursor, and mature IL-1β/Tubulin. The data are presented as the means ± SEMs. ^##^ *P* < 0.01, ^###^ *P* < 0.001 vs. the normal group; ^**^ *P* < 0.05, ^**^ *P* < 0.01, ^**^ *P* < 0.001 vs. the triptolide control.

AML12 cells are a type of liver cell line derived from mouse hepatocytes and retain many of the functional characteristics of primary hepatocytes, including the ability to metabolize drugs and produce proteins typically associated with liver function. So we used AML12 cells treated with triptolide to mimic an *in vitro* DILI model. We subsequently assessed the expression of pyroptosis-related proteins in the presence and absence of PEITC. Our findings indicated that the expression and activation levels of NLRP3, Caspase-1, GSDMD, and IL-1β were increased in AML12 cells following stimulation of triptolide. However, the addition of PEITC reduced the expression and activation of these proteins (Figure 6).

**FIGURE 7:**
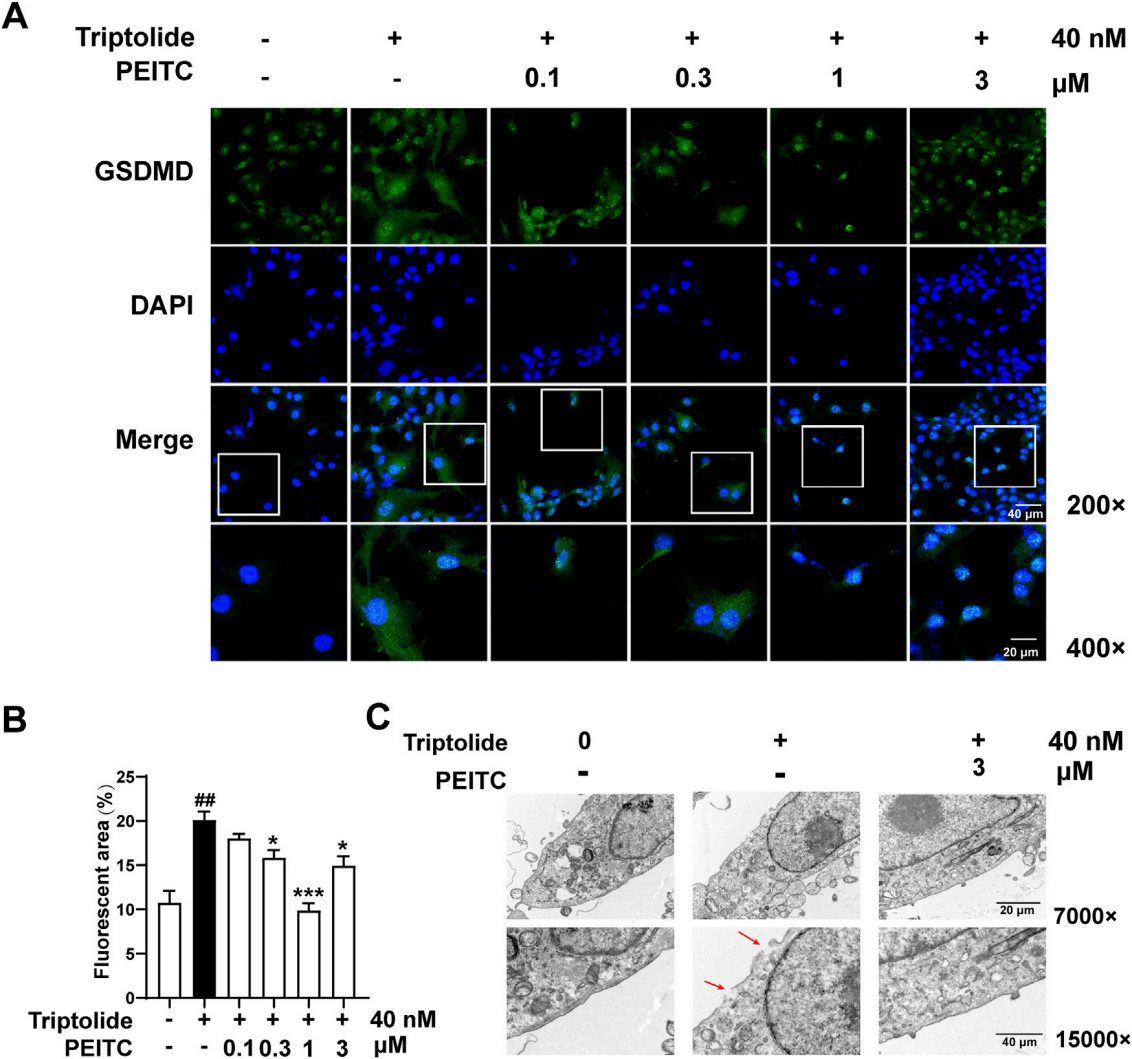
PEITC inhibited pyroptosis in AML12 cells treated with triptolide *in vitro*. **(A)** Representative transmission electron micrographs of AML12 cells from each group. **(B)** Immunofluorescence staining of GSDMD. **(C)** Immunofluorescence quantification of GSDMD. The data are presented as the means ± SEMs. ###*P* < 0.001 vs. the normal group, ***P < 0.001 vs. the triptolide control.

Additionally, our immunofluorescence analysis demonstrated that PEITC effectively inhibited the expression and localization of the key pyroptotic protein GSDMD in AML12 cells (Figure 7A). The typical characteristics of pyroptosis observed in AML12 cells following triptolide treatment included cell swelling, rupture of the cytoplasmic membrane, and the formation of intracellular vacuoles. In contrast, the morphology of AML12 cells treated with PEITC was restored, exhibiting a more intact plasma membrane and a reduction in intracellular vacuoles (Figure 7B).

## Discussion

Our study demonstrates that triptolide induces DILI with pronounced pyroptosis in hepatocytes in both *in vivo* studies and *in vitro* experiments. Conversely, PEITC, a natural compound found in cruciferous vegetables, exhibits a substantial protective effect against this liver injury. PEITC alleviates hepatic lesions, restores normal liver architecture, reduces serum transaminase levels in mice, and reestablishes normal hepatic metabolic function, underscoring its potential to mitigate DILI. This finding is pivotal for clinical applications, as triptolide, acetaminophen, and numerous chemotherapeutic agents that may induce hepatic injury possess unique pharmacological profiles, including potent anti-inflammatory, immunosuppressive, anti-tumor, and anti-fibrotic properties. These characteristics render them indispensable in specific therapeutic settings despite their hepatotoxicity, which constrains their clinical utility. Intensive research is underway to develop analogs or formulations that preserve these therapeutic benefits while minimizing toxicity.

Triptolide is one of the main active components of traditional Chinese medicine *Tripterygium wilfordii*, which has been widely used for its significant anti-cancer effects and the clinical treatment of systemic lupus erythematosus and rheumatoid arthritis. UHPLC-ESI-QTOF-MS method was used to observe the effects of *Tripterygium wilfordii* on serum and liver metabolites of mice. A total of 30 metabolites were identified, of which 29 were associated with toxicity severity. These metabolites are involved in a variety of metabolic pathways, including glutathione metabolism, tricarboxylic acid cycle (TCA cycle), purine metabolism, glycerophospholipid metabolism, *etc*. (Zhao et al., 2018). Among them, the study confirmed that triptolide can induce oxidative stress in HL7702 cells and HepG2 cells in mice, rats and human normal liver, significantly reduce superoxide dismutase (SOD) and Glutathione peroxidase (GSH-Px) activities, and significantly increase malondialdehyde (MDA) levels. In addition, it induces lipid peroxidation, increase autophagy activity, and inhibit Notch1, thereby increasing oxidative stress (Yang et al., 2012; Xu et al., 2019; Wei et al., 2019; Shen et al., 2019; Liu et al., 2013). The clinical use of triptolide typically begins with low doses; however, its therapeutic index is very narrow. As the dose increases beyond a certain threshold, symptoms of toxicity may emerge suddenly. The most prominent reaction associated with this acute poisoning is damage to the digestive system, particularly affecting the liver. The model used in this experiment simulates a state of acute poisoning induced by triptolide administration. In our study, triptolide administration resulted in hepatic injury characterized by a range of biochemical, and histological changes in mice (Figures 1, 2). Liver cell swelling, necrosis and inflammatory infiltration are observed. In addition, there was a significant release of cellular contents, including inflammatory molecules. NLRP3 inflammasome and caspases activation are also detected. All these data indicate that pyroptosis, a unique type of cell death, is widely occurring in the liver after triptolide is given to mice.

Pyroptosis represents a type of programmed cell death characterized by the lysis of cells and is facilitated by proteins that create pores, which are referred to as gasdermins. The family of gasdermins includes GSDMA, GSDMB, GSDMC, GSDMD, GSDME, and GSDMF, which is also known as PJVK or DFNB59. With the exception of PJVK, other gasdermin family members contain both C-terminal and N-terminal domains (Cavell et al., 2012). Gasdermins has a lipophile n-terminal domain with inherent pore-forming capabilities, as well as a C-terminal domain connected by a linker subregion. In the resting state, the C-terminal domain inhibited the pore-forming activity of the N-terminal domain. However, specific proteases are activated in response to different signals, cutting this junction region and separating the n-terminal and C-terminal domains, thus easing intramolecular inhibition. After lysis, the n-terminal domain is transferred to the plasma membrane, where it interacts with acidic phospholipids (such as inosine phosphate) on the cytoplasmic side of the membrane. N-terminal domains subsequently oligomerize and form annular pores in the plasma membrane, leading to cell lysis and pyroptosis. (Dayalan Naidu et al., 2018). In the canonical pathway, protein complexes including NLRP1, NAIP, NLRC4, AIM2, NLRP3, and Pyrin activate and cleave procaspase-1, producing active caspase-1. This active caspase-1 then processes GSDMD, producing an N-terminal truncated form (GSDMD-N) with pore-forming activity that ultimately leads to the release of the pro-inflammatory cytokines IL-1β and IL-18, which trigger an inflammatory response (Gupta et al., 2014; Wang et al., 2022). Alternatively, In the non-canonical pathway, lipopolysaccharide (LPS) can directly bind and activate caspases 11, 4, or 5, which subsequently cleave GSDMD to produce GSDMD-N (Wang et al., 2023). Activation of caspase-11/4 has been observed in the livers of both mice and patients with alcoholic fatty liver disease (HA), leading to hepatocyte pyroptosis via cleaved GSDMD (Zhao et al., 2018). Our previous research demonstrated that PEITC has a protective effect against acute immune-mediated liver injury induced by Concanavalin A and chemical liver injury caused by CCl_4_, primarily through direct interaction with GSDMD. Based on these findings, we further evaluated its effects on DILI in this study.

PEITC is a small-molecule compound derived from edible cruciferous vegetables, known for its antibacterial, anti-inflammatory, antioxidant, and anticancer properties (Coscueta et al., 2022). In a clinical trial of PEITC for managing prostate hyperplasia, we observed a reduction in serum transaminase levels among elderly patients. Additionally, PEITC significantly stimulates the reverse cholesterol transport pathway by regulating key proteins such as peroxisome proliferator activated receptor gamma (PPARγ), liver-X-receptor α (LXR-α), ATP binding cassette subfamily A member 1 (ABCA1), scavenger receptor A (SR-A1), cluster of differentiation 36 (CD36) 1, and NF-κB, thereby reducing lipid accumulation and inflammatory responses. Notably, PEITC supplementation has been shown to decrease lipid accumulation in the liver and mitigate atherosclerotic plaque formation in the aorta (Gwon et al., 2020). In this study, we found that PEITC significantly ameliorated liver damage induced by triptolide in mice (Figures 1C,E). During the experiment, we observed that the female representative liver of the triptolide group appears smaller and whiter than the one from the male. However, there are no significant differences in the parameters, such as body weight changes, serum transaminase concentrations, and pathological sections, between male and female mice. It has been reported that female mice experience more pronounced liver damage than male mice during acute liver injury induced by triptolide. Which may be attributed to the influence of estradiol in regulating macrophage-mediated inflammation and hepatocyte function (Zhang et al., 2024). These issues may be investigated in future studies. The activities of ALT, AST, and GGT, which are considered highly sensitive and specific biomarkers for liver toxicity, were markedly reversed following PEITC administration in mice with liver injury. Moreover, PEITC effectively restored the metabolic function of liver substances in a dose-dependent manner compared to the control group (Figure 2). It has been reported that olive oil, particularly its bioactive compounds such as monounsaturated or polyunsaturated fatty acids, may provide a certain level of hepatoprotection (Amamou et al., 2015; Albrahim et al., 2022). However, in our pilot experiment, we set up an olive oil solvent control and did not observe any protective effect on Triptolide-induced liver injury (data not shown). During this experiments, the equivalent volume of olive oil was also administered to the mice in the normal control and the Triptolide-treated only group. Livers from mice treated with Triptolide sustained notable damage (Figures 1C,E, 2). Consequently, it appears that PEITC was primarily responsible for the observed liver protective effects in this study.

The dose-dependent effects of phenethyl isothiocyanate (PEITC) on liver injury can be attributed to multiple mechanisms that vary with the concentration of PEITC administered. At low concentrations, PEITC has been shown to enhance the activity of antioxidants, such as glutathione peroxidase and superoxide dismutase, through the activation of the Nrf2 pathway. Additionally, it suppresses the expression of pro-inflammatory cytokines (e.g., TNF-α, IL-6) and inhibits the nuclear factor kappa B (NF-κB) signaling pathway (Coscueta et al., 2022). The presence of intracellular reactive oxygen species and inflammatory factors, such as IL-1β, suggests the occurrence of pyroptosis, a specific form of cell death, in hepatocytes following acute liver injury.

We further investigated whether PEITC could protect hepatocytes and the liver by inhibiting triptolide-induced pyroptosis. Compared to the model group, the PEITC-treated group exhibited reduced levels of pyroptosis-related proteins in liver tissues across all experimental groups (Figure 3A). Western blot and immunohistochemistry analyses demonstrated that PEITC significantly decreased the expression of pyroptosis-related proteins in the liver tissue of mice with triptolide-induced liver injury (Figures 4, 5). The clear dose-response relationship (5–20 mg/kg) *in vivo* indicates that PEITC may directly affect signaling pathways associated with pyroptosis, thereby protecting liver cells.

The therapeutic significance of PEITC in this context lies in its ability to modulate key signaling pathways associated with pyroptosis, particularly the NLRP3 inflammasome pathway. In Figure 6, it was shown that by inhibiting NLRP3 oligomerization and subsequent caspase-1 activation, PEITC effectively prevented the cleavage of GSDMD into its active form. PEITC also downregulated the expression of pro-inflammatory cytokines IL-1β, which are released during pyroptosis. By reducing the levels of these cytokines, PEITC helped attenuate the inflammatory response that often accompanies liver injury and cell death. As a form of programmed cell death, pyroptosis is characterized by distinct morphological features. In AML12 hepatocytes treated with triptolide, electron microscopy revealed numerous vesicle formations, membrane pore formation, membrane rupture, and cytoplasmic leakage. Following PEITC treatment, the morphology of AML12 cells showed significant improvement (Figure 7). These unique phenomenon confirmed PEITC’s potential as a therapeutic agent in the treatment of DILI, particularly in cases involving pyroptosis-related liver injury. By targeting the underlying processes that drive cell death and inflammation, PEITC offers a novel approach to protecting hepatic function and promoting recovery in patients suffering from drug-induced liver toxicity. Further research into these mechanisms can help refine therapeutic strategies and solidify the role of PEITC in liver health.

## Conclusion

In summary, PEITC demonstrates significant therapeutic potential in the treatment of drug-induced liver injury (DILI), particularly in cases involving hepatotoxic agents such as triptolide. The evidence highlights PEITC’s ability to mitigate liver damage through mechanisms that include the reduction of plasma transaminase, inhibition of pro-inflammatory IL-1beta, and suppression of pyroptotic cell death. These multifaceted actions position PEITC as a promising candidate for the management of liver injury caused by hepatotoxic substances, offering a novel approach to minimizing liver damage and promoting recovery.

Moving forward, future research should delve into the exploration of combination therapies that could enhance the protective efficacy of PEITC. Investigating synergistic effects with other hepatoprotective agents or integrating PEITC into existing treatment regimens may yield improved therapeutic outcomes for patients affected by DILI. Additionally, further studies are warranted to elucidate the precise molecular mechanisms underlying PEITC’s protective effects and to establish optimal dosing strategies that maximize its benefits while minimizing any potential risks. Given that PEITC has entered clinical trials in both the United States and China, it will be crucial for translating these findings into effective therapeutic interventions and for evaluating PEITC’s role within broader strategies for managing hepatotoxicity.

## Data Availability

The raw data supporting the conclusions of this article will be made available by the authors, without undue reservation.
